# Recurrent Mesenteric Ischemia from Celiomesenteric Trunk Stenosis

**DOI:** 10.7759/cureus.2751

**Published:** 2018-06-06

**Authors:** Atul Ratra, Samuel Campbell

**Affiliations:** 1 Texas Tech University Health Sciences Center, Lubbock, USA; 2 Department of General Surgery, Texas Tech University Health Sciences Center, Lubbock, USA

**Keywords:** mesenteric ischemia, celiomesenteric trunk, stenosis, endovascular revascularization, open surgical revascularization

## Abstract

A common origin of the celiac trunk and superior mesenteric artery (SMA), also termed as celiomesenteric trunk (CMT), is a rare occurrence. We report a rare case of symptomatic CMT stenosis requiring multiple interventions. The patient underwent an initial superior mesenteric artery bypass graft but required a subsequent endoluminal intervention. We present this case for the rarity of CMT stenosis and its potential to cause recurrent mesenteric ischemia. The treatment outcome in this patient suggests that revascularization of the SMA alone can result in adequate perfusion of the entire mesenteric bed and resolve symptoms of mesenteric ischemia including weight loss and food avoidance.

## Introduction

The arterial supply to the abdominal viscera arises from the celiac axis, superior mesenteric artery (SMA), and inferior mesenteric artery (IMA). Hemodynamically significant atherosclerosis in at least two of the three vessels is the most common cause of chronic mesenteric ischemia [1-3]. Other causes such as celiac artery compression syndrome in which the celiac artery gets compressed by the median arcuate ligament of the diaphragm have also been described [4].

While mesenteric atherosclerosis is common, patients with chronic mesenteric ischemia are rarely symptomatic due to the development of extensive collateral circulation over time. Stenosis of two of the three vessels is often required to manifest symptoms of chronic mesenteric ischemia [5]. Failure to treat could lead to serious complications including bowel infarction and death [6, 7].

A common celiomesenteric trunk (CMT) is a rare anatomic variation of the abdominal vasculature. Disease involving CMT is even rarer. Stenosis of the CMT can present with mesenteric ischemia as it can impair blood flow. We report a case of CMT stenosis causing recurrent mesenteric ischemia. The patient underwent extra-anatomic retrograde SMA bypass grafting that resulted in complete resolution of patient’s symptoms of weight loss, decreased appetite secondary to sitophobia, and dyspepsia. The patient underwent percutaneous angioplasty and stenting after recurrence of dyspepsia four years later. To our knowledge, this is the first reported case of CMT stenosis causing recurrent mesenteric ischemia despite surgical intervention and the first instance involving repair of CMT stenosis using both endovascular stenting and open graft repair modalities.

## Case presentation

A 79-year-old woman with a history of severe aortoiliac occlusive disease requiring a previous aorto-bi-iliac bypass graft presented in 2009 with a two-year history of chronic abdominal pain. Her abdominal pain was diffuse and postprandial in occurrence. She had associated symptoms of sitophobia (fear of food) and a weight loss of 87 pounds from 170 lbs to 83 lbs over a two-year period. At an outside hospital, the patient had workup performed for her symptoms over the previous two years that included an abdominal ultrasound, four esophagogastroduodenoscopies (EGDs), three colonoscopies, upper GI series and a non-IV contrast abdominal computed tomography (CT) scan – all with non-specific results.

**Figure 1:**
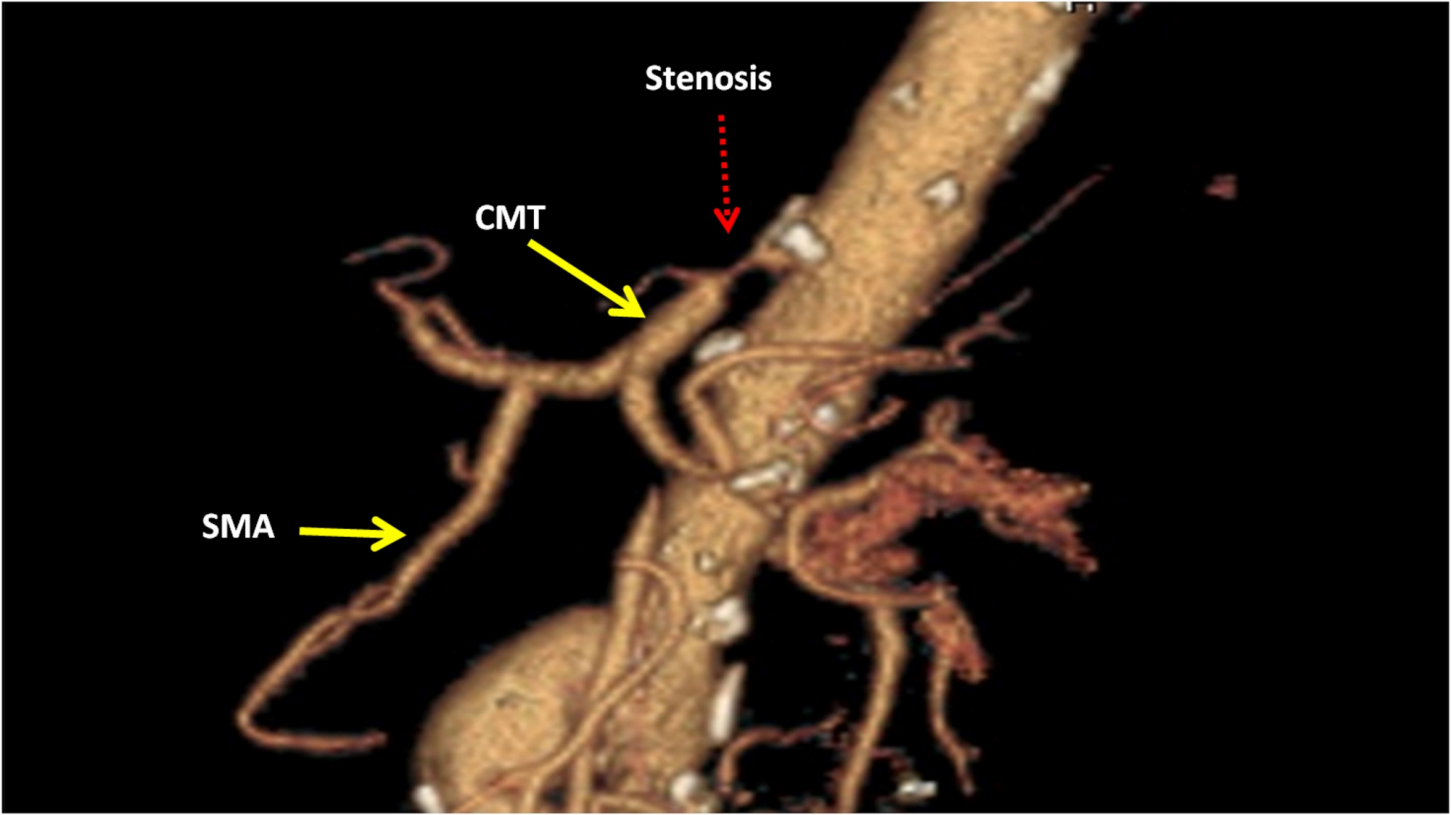
3D reconstruction of CT angiography of the abdomen showing rare CMT trunk with proximal stenosis. CMT: Celiomesenteric trunk; CT: Computed tomography; SMA: Superior mesenteric artery.

After referral, a duplex mesenteric arterial study revealed a peak systolic velocity (PSV) > 350 cm/sec within the celiac trunk indicating severe stenosis. She had reproduction of her abdominal pain after a postprandial challenge. CT angiography of the abdominal vessels revealed a common trunk of the superior mesenteric artery and celiac axis. The study confirmed a 90% stenosis of the celiomesenteric trunk (Figure 1). The IMA was also occluded. The aorto-bi-iliac bypass graft limbs were patent to the anastomotic site of the external iliac arteries. Based on the patient’s clinical presentation and diagnostic studies, a diagnosis of CMT ischemic syndrome was made.

**Figure 2:**
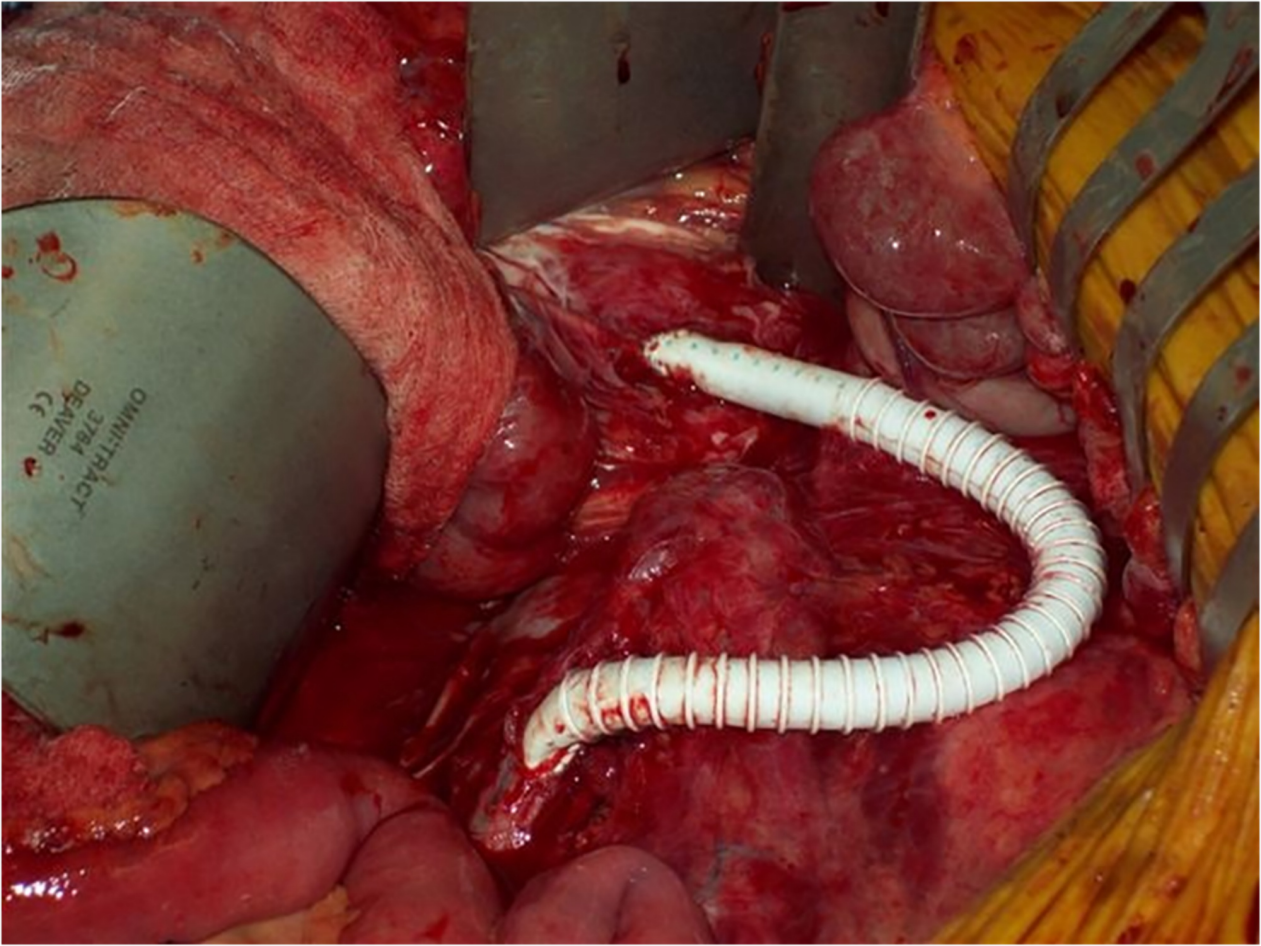
Intra-operative picture showing right iliac to SMA retrograde bypass graft placement. SMA: Superior mesenteric artery.

The first intervention occurred in 2009 when open bypass graft was the standard of care for mesenteric revascularization. The patient underwent an extra-anatomic right iliac to SMA retrograde bypass graft. The bypass was performed from the right limb of her aorto-bi-iliac bypass graft to the SMA with an 8 mm ringed Gore® Propaten® graft in an end-to-side fashion for both distal and proximal anastomosis (Figure 2). An end-to-side anastomosis to the SMA was adopted to enable forward flow to the SMA and retrograde flow to the celiac vessels. She had complete resolution of her symptoms and regained her body weight appropriately over a six-month period. She remained symptom-free for multiple years postoperatively.

The patient returned four years later with symptoms of postprandial pain and dyspepsia. No weight loss had occurred. Duplex arterial abdominal ultrasound showed severe stenosis of the CMT with elevated PSV of 461 cm/sec. Repeat CT angiography of the abdomen revealed dense calcific plaques throughout the native abdominal aorta. A 99% stenosis of the proximal celiomesenteric trunk was noted. The right iliac graft limb to SMA bypass graft was patent, however, the segment retrograde from the SMA anastomosis to the celiac vessels was occluded.

**Figure 3:**
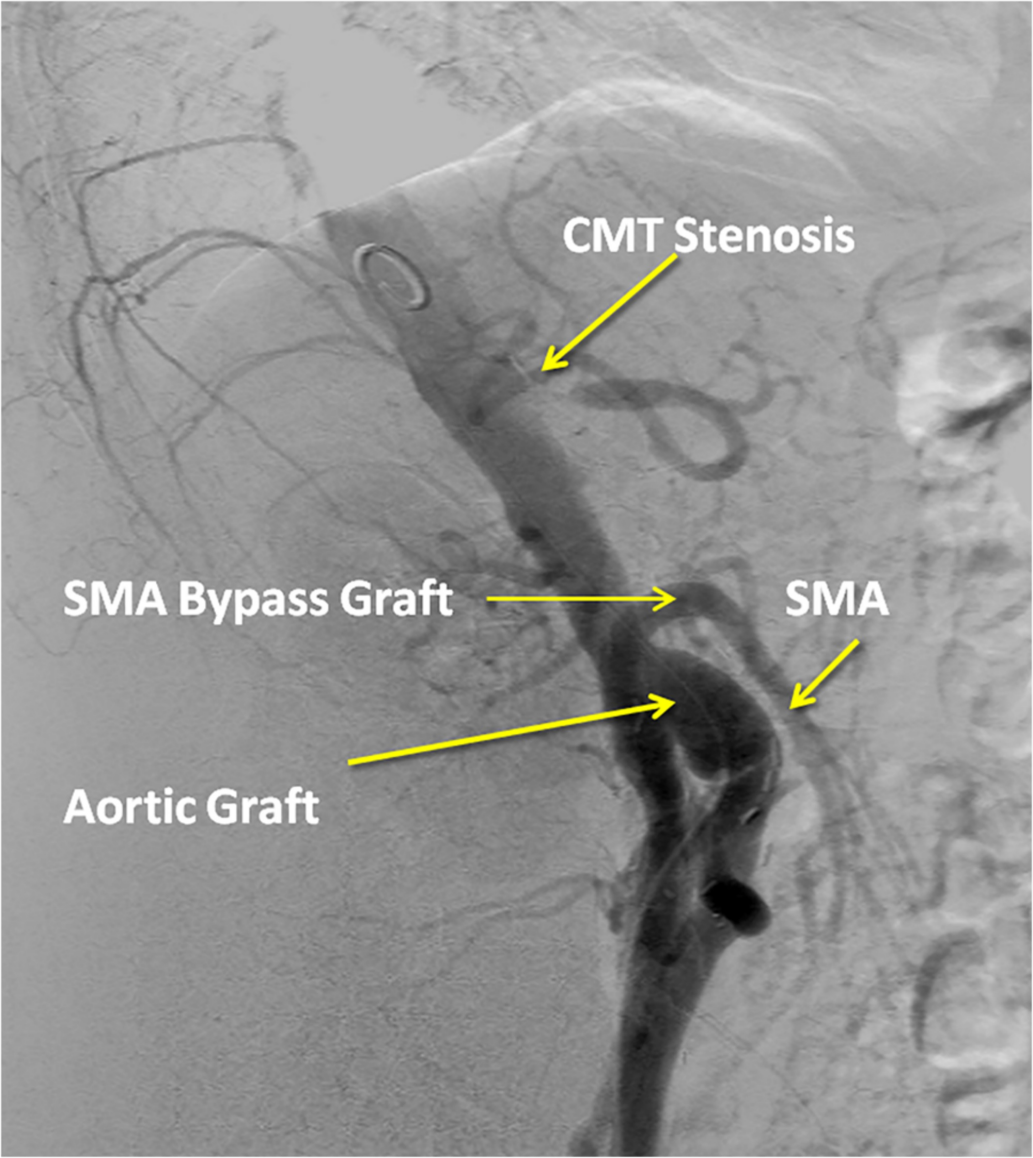
Aortogram showing proximal CMT stenosis but patent right iliac to SMA bypass graft. CMT: Celiomesenteric trunk; SMA: Superior mesenteric artery.

The patient underwent formal mesenteric angiography that confirmed a 99% stenosis of the proximal CMT and a patent right iliac limb to SMA bypass graft (Figure 3). She underwent a second surgical intervention with the placement of a 7 mm x 16 mm Atrium® stent in the proximal CMT via a retrograde femoral artery approach. The patient reported complete resolution of her abdominal complaints post-procedure. The patient has been followed at the clinic for the past five years and has remained symptom-free.

## Discussion

Splanchnic arteries arise from the primitive aorta in the fourth week of fetal development. During embryogenesis, visceral arteries develop from four roots that exit the dorsal abdominal aorta, which in the order of appearance are the gastric artery, hepatic artery, splenic artery and the SMA. These roots are thought to be initially joined by a longitudinal ventral anastomosis [8]. Under normal circumstances, a cleft forms between the third and fourth roots, which defines the separation between the celiac and superior mesenteric arteries. Failure of this cleft to form produces various anatomic variations including the formation of the extremely rare celiomesenteric trunk, which was the anatomic anomaly in our patient [9].

Reported incidence of CMT is between 0.5 and 2.7% of the general population [1, 4, 9, 10]. Pathologies involving CMT are very rare and include stenosis and aneurysms of the common trunk [4, 8]. Review of the literature reveals only six cases of CMT stenosis [1, 4, 10-13]. In the six case reports, one patient had an open bypass graft, two had percutaneous angioplasty/stenting, one underwent open surgical endarterectomy with patch-graft angioplasty, and one underwent open thrombectomy. One patient died before vascular intervention could be performed [13].

To our knowledge, this is the first reported case of recurrent symptomatic mesenteric ischemia due to CMT stenosis requiring repeated intervention. Additionally, it is the first reported case of open bypass grafting in a patient with CMT stenosis where the bypass graft originated from a pre-existing graft to create a bypass-to-bypass configuration rather than a bypass from a native vessel to provide retrograde CMT flow.

Atherosclerosis commonly develops at branch points within the vasculature. A common celiomesenteric trunk thus has a strong potential for development and progression of atherosclerosis along the trunk. Stenosis of the trunk is a serious pathological condition with profound clinical implications as it can impair blood supply to the majority of the gastrointestinal tract. Our patient had a resolution of postprandial pain and regained her normal weight post bypass to the SMA after the first intervention. Interestingly, she again became symptomatic with dyspepsia after the native SMA proximal to the SMA bypass became occluded. Such an occurrence seems to suggest that a patent SMA alone can provide adequate perfusion to the entire mesenteric distribution despite occlusion of other major mesenteric vessels. Targeting revascularization of SMA alone can lead to resolution of symptoms of postprandial pain and food avoidance. Also, we noted resolution of dyspepsia after endovascular stenting of the proximal CMT which suggests that revascularization of the celiac system provides relief from dyspepsia in patients with mesenteric occlusion.

Accurately identifying the existence of an anatomic vascular anomaly such as in this patient can thus help guide effective treatment approach for resolution of symptoms. In 2009, the standard of care for mesenteric revascularization was open bypass or endarterectomy [3, 6]. Endovascular approach has grown in practice over the years and is now considered the first line treatment for mesenteric revascularization. Endovascular technique is associated with lower mortality and lower costs compared to open surgical revascularization although with higher risk of re-stenosis and symptom recurrence in the long term [14]. Our case illustrates that both approaches provide adequate symptom relief for multiple years.

## Conclusions

In conclusion, the potential for anatomic variation in mesenteric vessels must be considered in patients presenting with symptomatic chronic mesenteric ischemia. Both endovascular repair and open surgical repair with bypass grafting are viable surgical options for the treatment of mesenteric ischemia from CMT stenosis with good outcomes and immediate symptom relief. In-stent re-stenosis and failure of retrograde flow through a bypass graft present long-term challenges in the management of such patients.
